# Risk factors and nomogram for predicting mechanical ventilation in severe pneumonia

**DOI:** 10.3389/fmed.2025.1598952

**Published:** 2025-09-15

**Authors:** Yong-Jia Chen, Yu-Fei Zhang, Da-Lang Huo, Dan Luo, Wei-Wei Chen

**Affiliations:** ^1^Department of Hospital Infection Management, The First People's Hospital of Zunyi (The Third Affiliated Hospital of Zunyi Medical University), Zunyi, China; ^2^Department of Infectious Diseases, The First People's Hospital of Zunyi (The Third Affiliated Hospital of Zunyi Medical University), Zunyi, China; ^3^Department of Gastroenterology, The First People's Hospital of Zunyi (The Third Affiliated Hospital of Zunyi Medical University), Zunyi, China

**Keywords:** severe pneumonia, mechanical ventilation, nomogram, logistic regression, predictive model

## Abstract

**Background:**

Severe pneumonia often leads to acute respiratory failure requiring mechanical ventilation (MV), significantly increasing patient morbidity and mortality. Early prediction of MV requirement could optimize patient management and resource allocation. This study aimed to identify key risk factors and develop a practical nomogram model to predict the need for mechanical ventilation among patients with severe pneumonia.

**Methods:**

In this retrospective study, patients with severe pneumonia admitted between January 2021 and December 2024 were analyzed at a single tertiary institution. Patients were stratified based on the use of MV within 24 h of admission. Multivariable logistic regression identified independent predictors of MV, which were used to construct a nomogram. Model performance was evaluated via receiver operating characteristic (ROC) curves, bootstrap validation, calibration, and decision curve analysis (DCA).

**Results:**

A total of 216 patients were included, with 165 in the MV group and 51 in the non-MV group. Patients requiring MV were significantly older and demonstrated lower oxygenation index (OI), partial pressure of oxygen [p(O₂)], central venous oxygen saturation (ScvO₂), and procalcitonin (PCT) levels, along with higher partial pressure of carbon dioxide [p(CO₂)], alveolar-arterial oxygen gradient [p(A-a)O₂], and APACHE II scores (all *p* < 0.01). Age, OI, p(O₂), p(CO₂), and p(A-a)O₂ were independent predictors included in the nomogram. The model showed excellent discrimination (area under the ROC curve, AUC = 0.819), calibration (concordance index, C-index = 0.805), and substantial clinical utility.

**Conclusion:**

This retrospective study suggests that age, OI, p(O₂), p(CO₂), and p(A-a)O₂ could help predict MV in severe pneumonia. The proposed nomogram might offer good predictive accuracy, calibration, and clinical utility, potentially aiding early risk stratification. Prospective multicenter validation is needed to confirm its generalizability and clinical utility.

## 1 Introduction

Severe pneumonia remains a major cause of morbidity and mortality worldwide, particularly among critically ill patients requiring intensive care unit (ICU) admission. It is frequently complicated by acute respiratory failure, often necessitating mechanical ventilation (MV) as a life-saving intervention. However, MV is associated with prolonged ICU stays, increased healthcare costs, and elevated risks of ventilator-associated complications, including ventilator-associated pneumonia (VAP), barotrauma, and ventilator-induced lung injury (VILI). Given these substantial clinical implications, early identification of patients at high risk for MV is crucial for optimizing resource utilization, enabling timely intervention, and potentially improving clinical outcomes (1–3).

Several studies have investigated risk factors associated with the need for MV in severe pneumonia, highlighting advanced age, comorbidities, and laboratory indicators of systemic inflammation or impaired gas exchange as key contributors (4, 5). However, existing predictive models are often limited by complexity, heterogeneity in patient populations, and inconsistent predictive performance. Therefore, there is an increasing demand for a clinically practical, accurate, and individualized risk stratification tool to predict MV requirements in patients with severe pneumonia (6–8). Nomogram models have gained widespread recognition as effective tools for individualized risk assessment across various clinical contexts. They offer a graphical representation of predictive algorithms that integrate multiple variables into an intuitive scoring system. Compared with traditional risk scoring methods, nomograms enhance predictive accuracy by quantitatively incorporating patient-specific factors. In recent years, nomograms have been successfully applied to predict adverse outcomes in respiratory conditions such as acute respiratory distress syndrome (ARDS), COVID-19-related pneumonia, and sepsis-induced respiratory failure. However, their application in predicting MV requirements specifically in patients with severe pneumonia remains insufficiently explored.

This study aims to systematically analyze the risk factors associated with MV requirement in severe pneumonia patients and develop a nomogram model to predict the probability of MV initiation. Additionally, the model’s performance will be validated through internal validation measures to assess its discrimination, calibration, and clinical utility. Ultimately, this approach may contribute to improved patient outcomes by enabling timely interventions, reducing unnecessary MV exposure, and mitigating the complications associated with prolonged mechanical ventilation.

## 2 Methods

### 2.1 Study design

This retrospective study evaluated patients with severe pneumonia treated at our institution between January 2021 and December 2024. A total of 216 patients meeting the predefined diagnostic criteria for severe pneumonia were enrolled. Patients were stratified based on the administration of mechanical ventilation within the first 24 h of hospital admission into two distinct cohorts: those who received mechanical ventilation (*n* = 165) and those who did not (*n* = 51). The study design, methodology, and analytical protocols were developed in accordance with the Strengthening the Reporting of Observational Studies in Epidemiology (STROBE) guidelines (9). Informed consent was obtained from all subjects. This study was rigorously reviewed and approved by our hospital’s ethics committee (No. KY240157), adhering to all applicable guidelines and regulations. It was conducted in strict compliance with the Declaration of Helsinki’s ethical standards for research involving human subjects. All study designs, executions, and reports maintained high ethical standards. To ensure privacy, data confidentiality was strictly upheld, and personal identifiers were anonymized prior to analysis.

### 2.2 Inclusion and exclusion criteria

The study retrospectively enrolled patients who met the following inclusion criteria:

1. Adult patients (≥18 years) with a confirmed diagnosis of severe pneumonia, established based on recognized diagnostic criteria such as those outlined by the American Thoracic Society/Infectious Diseases Society of America (ATS/IDSA).
2. Availability of comprehensive clinical data, including demographic characteristics, laboratory test results, imaging findings, and detailed records of respiratory support interventions.
3. Documented mechanical ventilation status within the first 24 h of hospital admission.

Patients were excluded from the study if they met any of the following exclusion criteria:

1. Patients with an alternative primary diagnosis or underlying respiratory condition (e.g., active pulmonary tuberculosis, advanced chronic interstitial lung disease, or primary lung malignancy) that could confound the diagnosis and management of severe pneumonia.
2. Individuals who received mechanical ventilation prior to hospital admission or in settings outside of our institution.
3. Patients with incomplete or missing clinical, laboratory, or imaging data that precluded a comprehensive analysis.
4. Cases in which treatment decisions were influenced by the presence of do-not-resuscitate (DNR) orders or other advanced directives limiting therapeutic interventions, as these factors may affect the decision to initiate mechanical ventilation.

### 2.3 Data collection and outcome measures

Clinical data were retrospectively extracted from the hospital’s electronic medical records. The collected parameters included demographic characteristics (gender and age) and a series of physiological and laboratory variables measured at admission. Specifically, arterial blood gas parameters such as partial pressure of oxygen [p(O₂)], partial pressure of carbon dioxide [p(CO₂)], alveolar-arterial oxygen gradient [p(A-a)O₂], and the oxygenation index (OI) were recorded. Additionally, arterial lactate levels at admission and at 6 h post-admission were documented. Other parameters included central venous oxygen saturation (ScvO₂), standard base excess (BE), and inflammatory biomarkers, namely procalcitonin (PCT), D-dimer, and C-reactive protein (CRP). The severity of illness was assessed using the Acute Physiology and Chronic Health Evaluation II (APACHE II) score. Furthermore, the utilization of vasoactive agents following fluid resuscitation was noted as an important therapeutic variable.

### 2.4 Statistical analysis

Data were processed using R (version 3.5.2), with key packages including mice, glm, predict, ResourceSelection, and rms. Normality of the continuous variables was assessed using the Kolmogorov–Smirnov test. Variables following a normal distribution were expressed as mean ± standard deviation (x ± s) and compared between groups using the independent-samples *t*-test. For non-normally distributed continuous data, the median and interquartile range [M (P25, P75)] were calculated, with group comparisons performed using the Wilcoxon rank-sum test. Categorical variables were presented as counts and percentages, and intergroup differences were evaluated using the chi-square test.

Multivariable logistic regression was employed to identify independent risk factors associated with the requirement for mechanical ventilation and to construct a predictive model, which was subsequently illustrated by a nomogram. The entire dataset was used as the training set, from which 30% of the cases were randomly and non-repeatedly selected to form an internal validation cohort. Model performance was primarily assessed by the receiver operating characteristic (ROC) curve and the area under the curve (AUC), and the predictive ability of models built with different parameter sets was compared. The goodness-of-fit of the model was evaluated using the Hosmer–Lemeshow test, and calibration curves were generated for both the training and validation sets. Finally, decision curve analysis (DCA) was conducted to assess the clinical utility of the predictive model. Statistical significance was set at *α* = 0.05.

To evaluate potential multicollinearity among predictor variables included in the multivariable logistic regression model, we performed two diagnostic assessments. First, pairwise Pearson correlation coefficients were calculated to examine linear associations between continuous variables. Second, the variance inflation factor (VIF) was computed for each predictor. A VIF value exceeding 5 was considered indicative of moderate collinearity, while a value greater than 10 suggested serious multicollinearity. Variables with high collinearity were carefully examined, and decisions regarding their inclusion were based on clinical relevance and statistical independence.

## 3 Results

### 3.1 Baseline characteristics of severe pneumonia patients

A total of 216 severe pneumonia patients were divided into the non-MV group (*n* = 51) and MV group (*n* = 165) based on the need for mechanical ventilation within 24 h. The MV group was significantly older (median 73.00 vs. 64.00 years, *p* < 0.01) and exhibited notably lower PCT levels (median 2.21 vs. 10.16 μg/L, p < 0.01) and reduced ScvO₂ (0.70 ± 0.15 vs. 0.77 ± 0.10, *p* < 0.01). In addition, patients in the MV group showed a significantly higher alveolar-arterial oxygen gradient [p(A-a)O₂, median 158.62 vs. 106.71 mmHg, *p* < 0.01] and APACHE II scores (23.94 ± 8.06 vs. 21.18 ± 7.73, *p* < 0.01). Furthermore, the MV group had a lower oxygenation index (median 133.49 vs. 198.89 mmHg, *p* < 0.01), lower p(O₂), and higher p(CO₂) compared to the non-MV group (both *p* < 0.01). Other parameters, including CRP, D-dimer, lactate levels, and vasoactive drug usage, did not differ significantly between the groups (Table 1).

**Table 1:** Baseline characteristics of severe pneumonia patients: mechanical ventilation group vs. non-mechanical ventilation group.

Parameter	Non-MV group (*n* = 51)	MV group (*n* = 165)	*χ*^2^, *t*, or *Z*	*p*-value
Demographics and initial laboratory data				
Gender (M/F)	41/10	111/54	3.22	0.07
Age (years), median (IQR)	64.00 (53.00, 70.00)	73.00 (64.00, 81.00)	3.45^**^	<0.01
CRP (mg/L), median (IQR)	87.55 (60.69, 169.44)	101.00 (57.60, 200.50)	0.48	0.62
PCT (μg/L), median (IQR)	2.21 (0.63, 22.56)	10.16 (2.07, 38.44)	3.23	<0.01
D-dimer (mg/L), median (IQR)	2.67 (1.61, 7.47)	3.71 (2.11, 9.54)	1.77	0.17
Lac (mmol/L), median (IQR)	3.40 (2.21, 6.44)	5.67 (2.70, 7.42)	1.72	0.18
ScvO₂, mean ± SD	0.77 ± 0.10	0.70 ± 0.15	2.88	<0.01
Admission 6 h Lac (mmol/L), median (IQR)	2.47 (1.44, 7.54)	3.81 (1.85, 6.22)	1.12	0.27
Additional clinical parameters				
p(A-a)O₂ (mmHg), median (IQR)	106.71 (75.81, 144.20)	158.62 (97.03, 236.59)	2.50	<0.01
BE (mmol/L), median (IQR)	−7.83 (−12.57, −5.25)	−9.27 (−14.63, −1.44)	0.39	0.56
APACHE II score, mean ± SD	21.18 ± 7.73	23.94 ± 8.06	2.23	<0.01
OI (mmHg), median (IQR)	198.89 (160.87, 283.03)	133.49 (85.00, 183.34)	5.22	<0.01
p(O₂) (mmHg), median (IQR)	68.50 (57.29, 93.73)	63.45 (43.88, 73.34)	2.74	<0.01
p(CO₂) (mmHg), median (IQR)	30.90 (24.72, 35.02)	39.14 (30.90, 50.47)	4.29	<0.01
Therapeutic intervention				
Vasoactive drug usage, *n* (%)	36 (70.59%)	118 (71.52%)	0.02	0.90

** *p* < 0.01.

MV, mechanical ventilation; CRP, C-reactive protein; PCT, procalcitonin; Lac, lactate; ScvO₂, central venous oxygen saturation; p(A-a) O₂, alveolar-arterial oxygen gradient; BE, base excess; APACHE II, Acute Physiology and Chronic Health Evaluation II; OI, oxygenation index; p(O₂), partial pressure of oxygen; p(CO₂), partial pressure of carbon dioxide.

### 3.2 Multivariable logistic regression analysis

A multivariable logistic regression model was constructed with the requirement for mechanical ventilation as the dependent variable (yes = 1, no = 0). The following variables were incorporated as independent predictors: age, sex (male = 1, female = 0), PCT, ScvO₂, p(A-a)O₂, APACHE II score, OI, p(O₂), and p(CO₂). The analysis revealed that age, OI, p(O₂), p(CO₂), and p(A-a)O₂ were statistically significant independent predictors of mechanical ventilation requirement (all *p* < 0.05). Specifically, increasing age and p(CO₂) were associated with elevated odds of requiring mechanical ventilation, while higher OI and p(A-a)O₂ values were inversely related. Additionally, p(O₂) emerged as a significant predictor (Table 2). The regression coefficients (*β* values) along with the intercept (−0.847) are provided in Table 2. Based on these estimates, the final logistic regression model can be expressed as:


$$\begin{array}{l} \text{Model Equation}:\text{Logit}\;(P)=-0.847+0.054\times \mathrm{Age}–0.027\times \\ \mathrm{OI}+0.049\times p(O₂)+0.043\times p(\mathrm{CO}₂)–0.007\times p(A-a)O₂ \end{array}$$

**Table 2:** Logistic regression analysis for predicting mechanical ventilation requirement.

Factors	β-value	Standard error value	Wald value	OR value	95% CI for OR	*p*-value
Age	0.054	0.021	6.5	1.055	1.013–1.096	0.01
OI	−0.027	0.007	18.8	0.971	0.958–0.986	<0.001
p(O₂)	0.049	0.019	6.55	1.05	1.012–1.088	0.011
p(CO₂)	0.043	0.017	5.81	1.044	1.009–1.079	0.016
p(A-a) O₂	−0.007	0.003	7.25	0.993	0.988–0.998	0.007

*β*, regression coefficient; OR, odds ratio; CI, confidence interval; OI, oxygenation index; p(O₂), partial pressure of oxygen; p(CO₂), partial pressure of carbon dioxide; p(A-a) O₂, alveolar-arterial oxygen gradient.

### 3.3 Nomogram predictive model for mechanical ventilation

**Figure 1:**
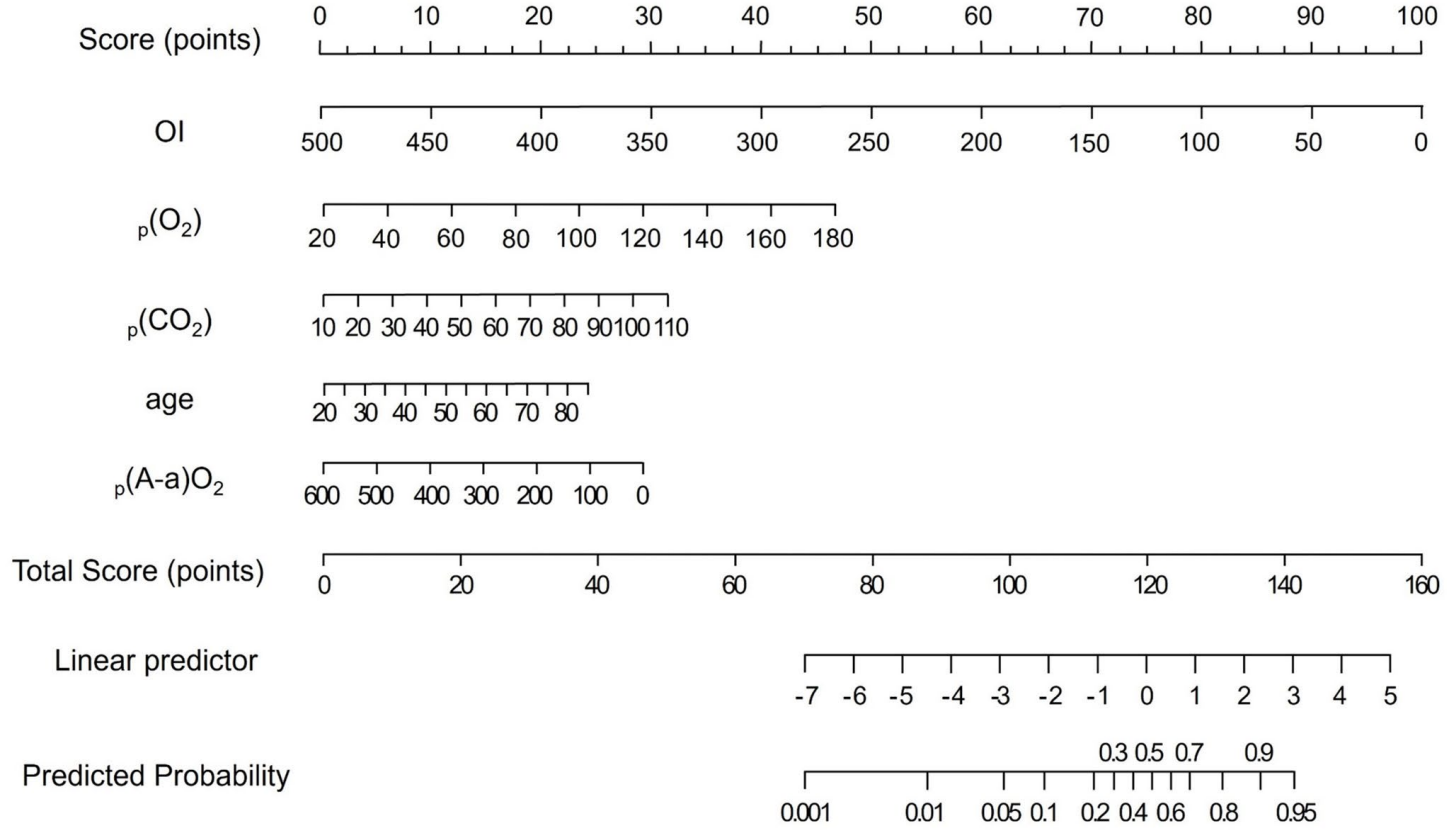
Nomogram predictive model for mechanical ventilation requirement in severe pneumonia patients.

Based on the multivariable logistic regression analysis, five independent predictors were identified as significantly associated with the need for mechanical ventilation. Utilizing these predictors, a nomogram was developed to estimate the probability of mechanical ventilation in severe pneumonia patients. In this nomogram, each predictor is represented by a dedicated axis with corresponding point scales. For an individual patient, the value of each predictor is mapped to its respective axis, and a vertical line is drawn upward to the point scale to determine the score for that variable. The individual scores are then summed to derive a total score, which is subsequently referenced on a probability scale at the bottom of the nomogram. Higher total scores correspond to a greater estimated risk of requiring mechanical ventilation (Figure 1).

### 3.4 Discriminative performance of the nomogram

**Figure 2:**
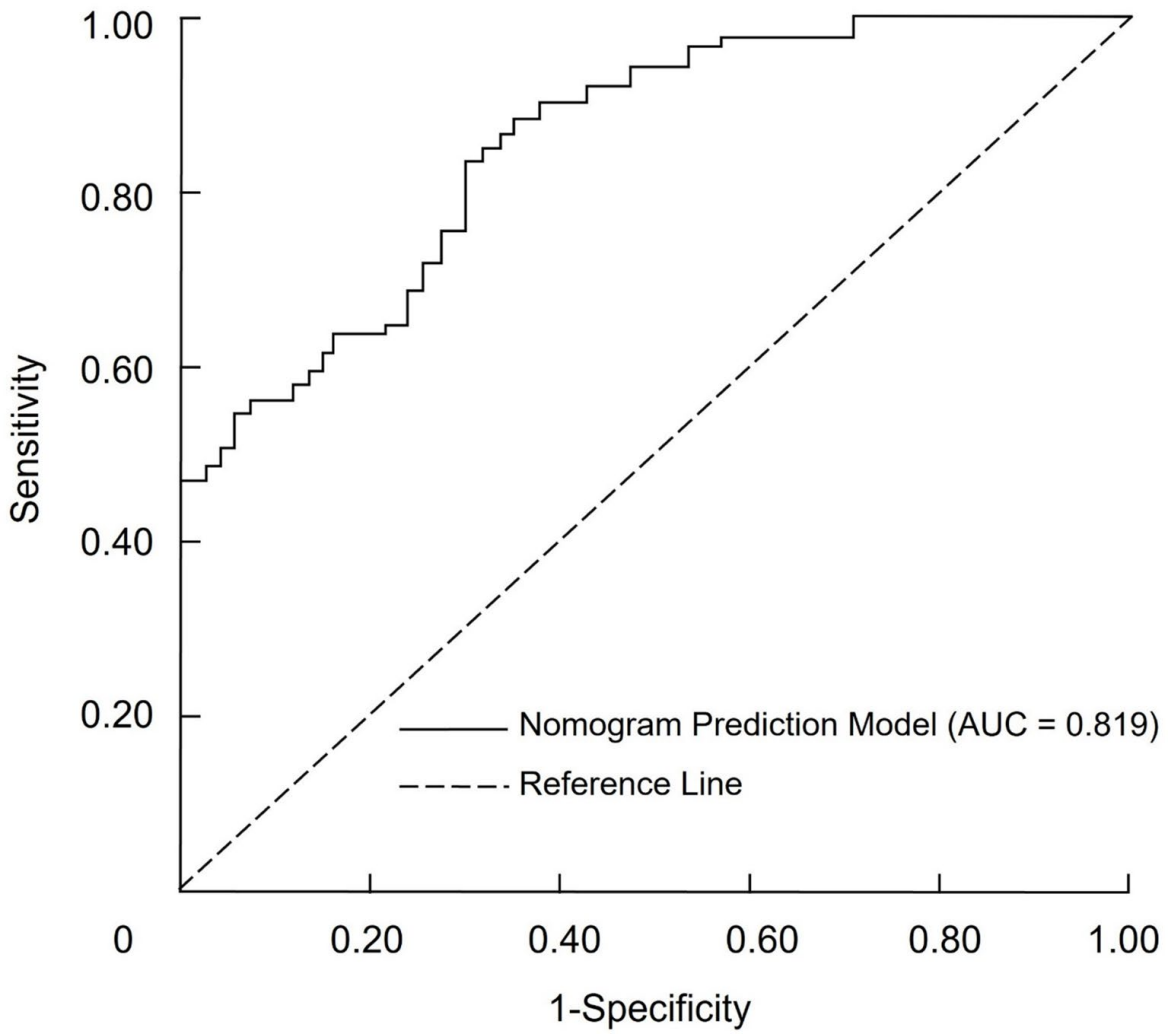
Receiver operating characteristic (ROC) curve of the nomogram for predicting mechanical ventilation in severe pneumonia patients.

The discriminative ability of the nomogram for predicting the requirement for mechanical ventilation was assessed using receiver operating characteristic (ROC) curve analysis. In this analysis, the total risk score derived from the nomogram served as the independent variable, while the binary outcome of mechanical ventilation requirement was the dependent variable. The model demonstrated an area under the ROC curve (AUC) of 0.819 (95% confidence interval, 0.776–0.906), indicating robust predictive accuracy. When the optimal cut-off point was determined using the maximum Youden index, the model yielded a sensitivity of 76.58% and a specificity of 88.15% (Figure 2).

### 3.5 Calibration of the nomogram predictive model

**Figure 3:**
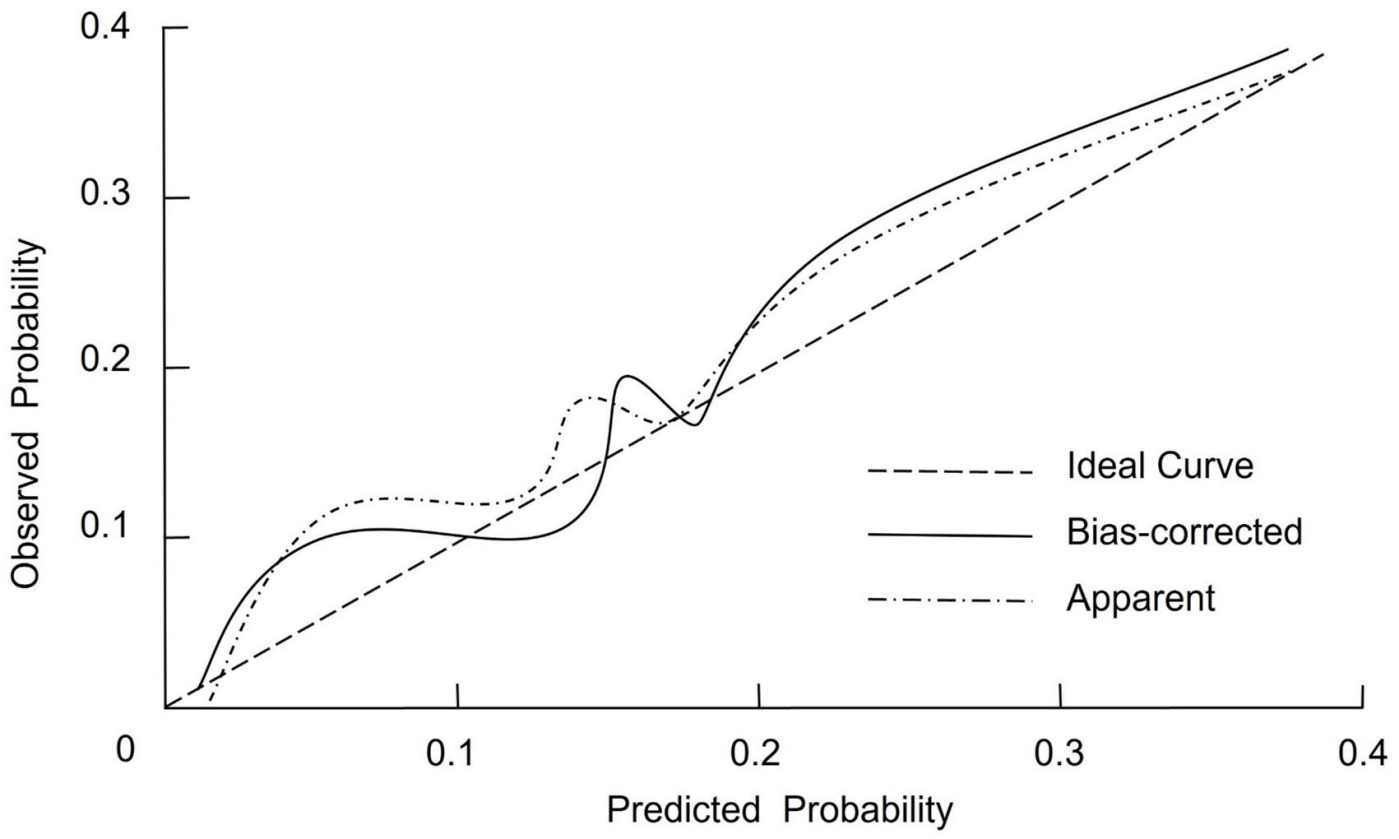
Calibration curve of the nomogram predictive model for mechanical ventilation in severe pneumonia patients.

The calibration of the nomogram was evaluated using internal validation via bootstrap resampling, with 1,000 iterations performed to assess model stability. The corrected concordance index (C-index) was 0.805 (95% CI, 0.769–0.851), indicating that the model has a high discriminative ability. Additionally, the Hosmer–Lemeshow goodness-of-fit test yielded a χ^2^ value of 2.856 (*p* = 0.893), suggesting that there is no significant difference between the predicted and observed outcomes. The calibration curve further corroborated these findings, demonstrating a close alignment between the predicted probabilities and the actual incidence of mechanical ventilation requirement (Figure 3).

### 3.6 Clinical utility of the nomogram predictive model

**Figure 4:**
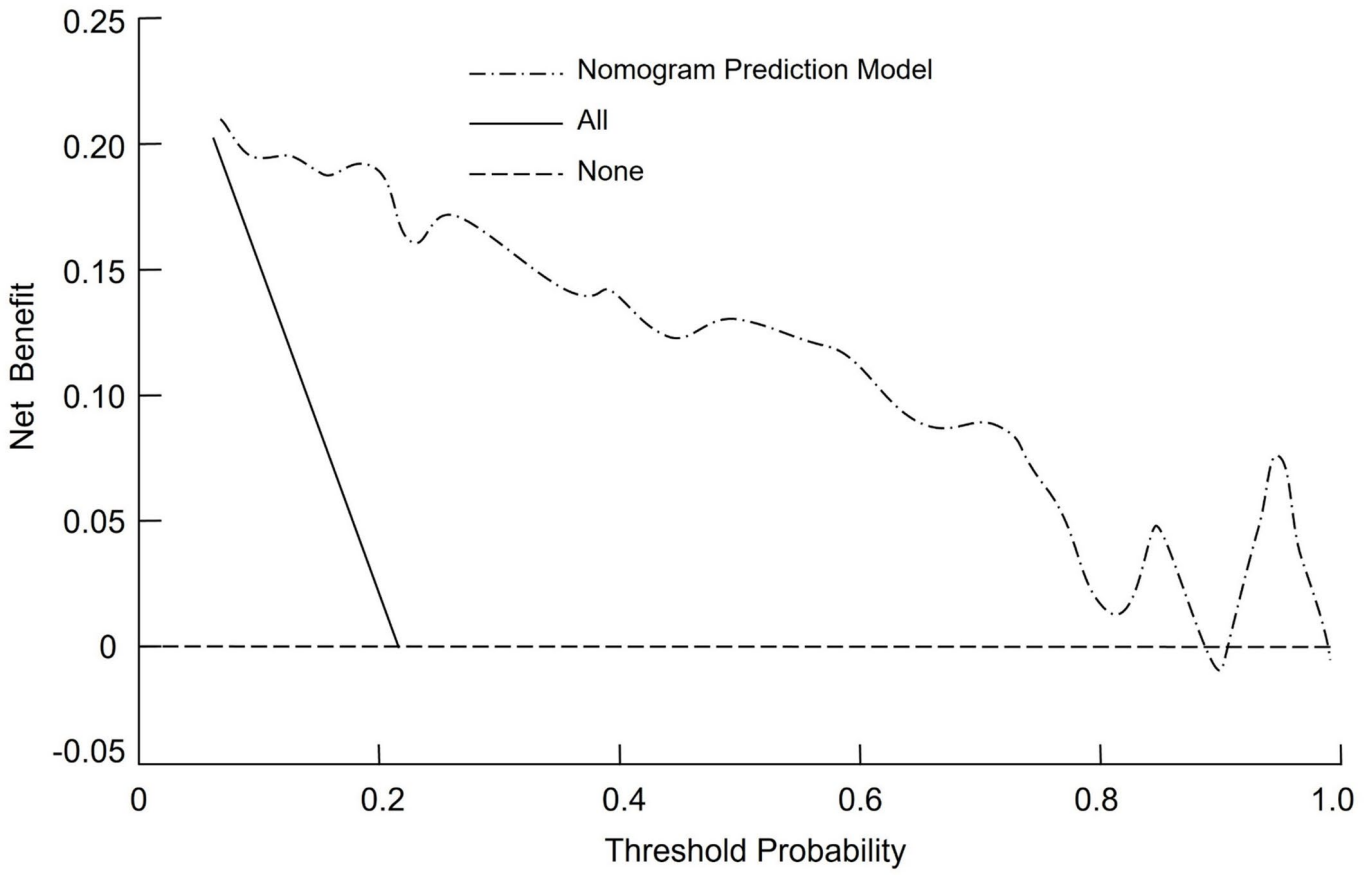
Decision curve analysis (DCA) of the nomogram predictive model for mechanical ventilation in severe pneumonia patients.

Decision curve analysis (DCA) was performed to assess the clinical utility of the nomogram in predicting the need for mechanical ventilation. In the DCA, the horizontal reference line represents the net benefit when no patients are treated with mechanical ventilation, yielding a net benefit of zero. Conversely, the sloping line corresponds to the strategy of treating all patients with mechanical ventilation, which results in a negative net benefit. The nomogram’s decision curve demonstrated a substantially higher net benefit across a range of threshold probabilities compared to these two extreme strategies (Figure 4).

### 3.7 Microbiological Etiology of severe pneumonia

Among the 216 patients included in this study, microbiological testing results were partially available. A total of 78 patients (36.1%) were confirmed to have typical bacterial infections, most commonly *Streptococcus pneumoniae*, *Klebsiella pneumoniae*, *Pseudomonas aeruginosa*, and *Staphylococcus aureus*. Viral pathogens, including influenza A/B, respiratory syncytial virus (RSV), and SARS-CoV-2, were identified in 51 patients (23.6%). Mixed bacterial–viral infections were present in 27 patients (12.5%). Atypical pathogens such as *Mycoplasma pneumoniae* and *Legionella pneumophila* accounted for 12 cases (5.6%). Fungal infections were rare (5 cases, 2.3%), involving *Candida albicans* or *Aspergillus* spp. In 43 patients (19.9%), no definitive pathogen could be identified due to either negative results or the absence of microbiological testing. These findings highlight the etiological heterogeneity in severe pneumonia and may partially explain biomarker variability (Table 3).

**Table 3:** Microbiological etiology of severe pneumonia patients (*n* = 216).

Pathogen category	Identified pathogen(s) example	No. of cases	Percentage (%)
Typical bacterial infections	*Streptococcus pneumoniae*, *Klebsiella pneumoniae*, *Pseudomonas aeruginosa*, *Staphylococcus aureus*	78	36.10%
Viral infections	Influenza A/B, SARS-CoV-2, RSV	51	23.60%
Mixed bacterial-viral infections	*Klebsiella* + Influenza A, etc.	27	12.50%
Atypical pathogens	*Mycoplasma pneumoniae*, *Legionella*	12	5.60%
Fungal infections	*Candida albicans*, *Aspergillus* spp.	5	2.30%
Unidentified/NO PATHOGEN DETECTED	—	43	19.90%
Total	—	216	100%

### 3.8 Assessment of multicollinearity

To evaluate potential multicollinearity among the predictors, pairwise Pearson correlation coefficients and variance inflation factors (VIFs) were calculated. As shown in Supplementary Table S1, moderate correlations were observed among certain arterial blood gas parameters, particularly between OI and p(A-a)O₂ (*r* = −0.62) and between OI and p(O₂) (*r* = 0.68). Importantly, no absolute correlation exceeded 0.70, and all VIF values were <5 (maximum VIF = 3.21 for OI), indicating the absence of severe multicollinearity. These findings support the statistical independence of the predictors included in the final multivariable logistic regression model. The reversal in the direction of association for p(A-a)O₂ between univariate and multivariable analyses is therefore more likely attributable to shared variance among respiratory parameters rather than to problematic collinearity or model misspecification.

### 3.9 *Post hoc* power analysis

To evaluate the adequacy of the sample size, a *post hoc* power analysis was performed for the five independent predictors included in the final logistic regression model: age, OI, p(O₂), p(CO₂), and p(A-a)O₂. The statistical power for each variable was calculated individually, and a weighted average power (with equal weighting across variables) was determined. The overall weighted post hoc power was 80.0%, meeting the conventional threshold for acceptable power. Most individual predictors demonstrated power levels at or near the conventional threshold of 80%, among the variables, OI exhibited particularly strong discriminatory power with an individual power exceeding 95%. These results support the adequacy of the current sample size in detecting meaningful associations between predictors and the requirement for mechanical ventilation.

## 4 Discussion

Severe pneumonia remains a major global health burden, associated with significant morbidity and mortality, particularly in cases progressing to respiratory failure requiring MV. Early identification of patients at high risk for MV can significantly improve clinical outcomes by enabling timely interventions and optimizing resource allocation in intensive care settings (10, 11). This study identified significant predictors of MV requirement in patients with severe pneumonia, including older age, lower OI, elevated p(CO₂), reduced p(O₂), and p(A-a)O₂. The developed nomogram model incorporating these factors demonstrated strong predictive performance (AUC = 0.819), good calibration (C-index = 0.805), and substantial clinical benefit in decision curve analysis. Collectively, these findings highlight the potential utility of the nomogram as an effective clinical tool for early identification and targeted intervention in severe pneumonia patients at high risk for mechanical ventilation.

Our findings revealed that older age significantly increased the risk of requiring mechanical ventilation. Age-related susceptibility in pneumonia has been attributed to immune senescence and chronic comorbidities. Elderly patients frequently exhibit diminished physiological reserves, including decreased pulmonary function, reduced respiratory muscle strength, impaired cough reflex, and compromised mucociliary clearance. These factors collectively exacerbate respiratory distress, increasing the likelihood of acute respiratory failure necessitating ventilatory support (12, 13). Additionally, advanced age is often associated with increased disease severity at presentation, reflected in elevated APACHE II scores observed in our MV group. We observed that elevated p(CO₂) was significantly associated with a higher likelihood of requiring mechanical ventilation. Hypercapnia is indicative of impaired alveolar ventilation, commonly due to increased airway resistance, respiratory muscle fatigue, or diminished respiratory drive, all frequently encountered in severe pneumonia. Furthermore, hypercapnia can exacerbate respiratory acidosis, potentiating further impairment of respiratory drive, respiratory muscle dysfunction, and hemodynamic instability, thus leading to a rapid deterioration in respiratory status. As a result, p(CO₂) serves as a valuable predictor, reflecting both respiratory muscle fatigue and inadequate gas exchange (14, 15).

A decreased OI emerged as another important predictor of MV. The oxygenation index, a composite parameter incorporating arterial oxygen tension and inspired oxygen fraction (FiO₂), is widely used to assess the severity of hypoxemia. A reduced OI typically indicates severe hypoxemia refractory to supplemental oxygen therapy, necessitating invasive ventilatory support. The lower OI observed in patients requiring MV likely reflects profound pulmonary involvement, significant ventilation-perfusion mismatch, and diminished alveolar recruitment capacity, which underscores the severity of lung injury in this patient population. Unexpectedly, we observed a lower median PCT level in the MV group compared to the non-MV group, despite traditionally higher PCT levels being linked to severe infection. This discrepancy could potentially be explained by heterogeneity in pathogen types or varying inflammatory responses among pneumonia subtypes (16, 17). It is plausible that some severe pneumonia cases with substantial respiratory compromise involve viral etiologies or atypical pathogens, conditions often associated with lower serum PCT concentrations. Thus, while elevated PCT is a well-established marker of bacterial infection severity, our results suggest that respiratory impairment severity, rather than systemic bacterial burden, predominantly dictates the need for mechanical ventilation. Additionally, the p(A-a)O₂, a well-established indicator of impaired pulmonary gas exchange, significantly differed between groups. Interestingly, while univariate analysis showed increased p(A-a)O₂ values in the MV group, the multivariable analysis indicated an inverse association with MV after adjusting for other predictors. This finding may reflect a complex interaction between p(A-a)O₂ and other respiratory parameters, such as OI and p(O₂). A potential explanation is that when considered independently, a higher p(A-a)O₂ denotes severe pulmonary impairment, yet once adjusted for OI and other gas-exchange parameters, its incremental predictive value diminishes or becomes inversely associated due to collinearity among parameters. Further research is necessary to clarify this unexpected relationship (18–20).

The predictive accuracy of the nomogram developed in our study was robust, as evidenced by an AUC of 0.819, a sensitivity of 76.58%, and a specificity of 88.15%. The high discrimination was confirmed via internal bootstrap validation, indicating a reliable and generalizable model. Additionally, calibration metrics, including a corrected concordance index of 0.805 and an excellent Hosmer–Lemeshow goodness-of-fit (*p* = 0.893), indicated the nomogram accurately estimates the probability of mechanical ventilation. The favorable decision curve analysis results further demonstrated that the model provides substantial clinical benefits by appropriately identifying high-risk patients who might benefit from early and aggressive respiratory interventions. Clinically, these results have important implications. Early recognition of high-risk patients allows for timely initiation of interventions, including appropriate respiratory support strategies such as noninvasive ventilation, high-flow nasal cannula, or early intubation, potentially mitigating progression to respiratory failure. Additionally, the use of such a predictive tool can optimize ICU resource allocation, ensuring that critical care beds and respiratory support equipment are reserved for those patients most likely to benefit.

The observed inverse association between PCT levels and MV requirement may reflect underlying etiological heterogeneity rather than a direct pathophysiological relationship. Our microbiological analysis revealed that a substantial proportion of patients had viral or mixed infections, which are known to elicit lower PCT responses despite severe respiratory compromise. This highlights the necessity of incorporating pathogen profiles when interpreting inflammatory biomarkers in predictive models. To ensure the integrity of our multivariable logistic model, we conducted a formal assessment of multicollinearity. Pearson correlation coefficients and VIFs confirmed no severe collinearity (all *r* < 0.70; VIF < 5), supporting the statistical independence of retained predictors. The reversal of p(A-a)O₂’s association between univariate and multivariate analyses likely stems from its shared variance with OI and p(O₂), rather than modeling bias. Model performance was internally validated using 1,000-bootstrap resampling, a widely accepted method for assessing predictive stability and minimizing overfitting, particularly when external data are unavailable. Lastly, *post hoc* power analysis confirmed adequate sample size, with a weighted average power of 80.0% and particularly strong discriminatory power for OI (>95%). Together, these findings reinforce the robustness, interpretability, and clinical applicability of our nomogram in early MV risk stratification.

Consistent with Niu’s study (21), who developed and externally validated machine-learning models for ICU mortality in severe pneumonia (AUC ≈ 0.74–0.77) and produced a logistic-based nomogram, our work likewise targets early risk stratification; however, we focused on the near-term need for MV within 24 h and achieved higher discrimination (AUC 0.819). Differences in endpoint definition, predictor sets (physiology-centric arterial blood gas indices vs. mixed clinical/laboratory features), and validation strategy (external vs. internal) likely account for performance differences and limit direct comparability. Viasus’ study (22) used inverse propensity score weighting to assess antibiotic de-escalation in severe CAP and found no deterioration in mortality or length of stay, with reduced prolonged intravenous therapy. While their study estimates treatment effects rather than builds prediction models, our nomogram can complement stewardship by flagging patients at higher MV risk upon admission—i.e., informing clinical triage in parallel with antimicrobial optimization. Yan’s study (23) identified age, APACHE II, and prolonged MV as risk factors for refeeding syndrome in ICU enteral-nutrition patients, underscoring that respiratory support intensity is tightly linked to downstream complications. In contrast, our model predicts the imminent need for MV using gas-exchange metrics (OI, pO₂, pCO₂, p(A-a)O₂), potentially enabling earlier escalation of respiratory support and mitigation of subsequent morbidity. Physiologically, lower oxygenation index and pO₂ and higher pCO₂ and p(A-a)O₂ plausibly reflect impaired oxygenation, ventilation failure, and increased shunt/ventilation–perfusion mismatch, which are proximal determinants of MV initiation. Age may capture diminished respiratory reserve and comorbidity burden. Inflammatory biomarkers (PCT) and global severity (APACHE II) did not remain independent after adjustment, suggesting that gas-exchange variables more directly mediate the decision for early MV. Potential residual confounding (practice patterns, sedation/vasopressor use) and selection bias inherent to retrospective design remain, reinforcing the need for prospective, multicenter validation and head-to-head comparisons with established severity indices.

This study has several limitations that warrant consideration. First, the retrospective, single-center design may limit the generalizability of our findings due to potential selection bias and center-specific clinical practices. Although we employed internal bootstrap resampling to mitigate overfitting and enhance model reliability, the absence of external validation remains a key limitation. Future prospective multicenter studies are essential to externally validate the model across diverse populations and healthcare settings. Second, due to the retrospective nature of data collection, patients with missing key clinical, laboratory, or imaging variables were excluded, and a complete case analysis was performed. While this approach ensured data integrity, it may have introduced selection bias. Third, although our model demonstrated adequate statistical power based on *post hoc* analysis, the relatively modest sample size may still limit the detection of less prominent predictors. Finally, this study did not evaluate longitudinal clinical outcomes following MV initiation. Future investigations should incorporate follow-up data to assess treatment responses, weaning success, and mortality, thereby providing a more comprehensive appraisal of clinical trajectories in patients requiring mechanical ventilation.

## 5 Conclusion

This retrospective study suggests that Age, OI, p(O₂), p(CO₂), and p(A-a)O₂ could be important predictors of MV in severe pneumonia. The derived nomogram might provide robust predictive accuracy, good calibration, and potential clinical utility. This tool could assist in the early identification of patients at higher risk, thereby supporting clinicians in making timely decisions to potentially optimize management strategies and patient outcomes. Further validation in large-scale, prospective, multicenter cohorts is essential to assess the model’s generalizability, refine its predictive performance, and determine its real-world applicability.

## Data Availability

The original contributions presented in the study are included in the article/Supplementary material, further inquiries can be directed to the corresponding author.
