# Supplementary material for: Risk factors and nomogram for predicting mechanical ventilation in severe pneumonia
Source: Front Med (Lausanne). 2025 Sep 15;12:1598952. doi: 10.3389/fmed.2025.1598952 (PMC12477012; doi:10.3389/fmed.2025.1598952)
Supplement: Supplementary file 1 [file Table_1.DOCX]

Supplementary Table S1. Pairwise Pearson (r) Correlation Matrix for the Five Candidate Predictors

| Variable | Age | OI | p(O₂) | p(CO₂) | p(A-a)O₂ |
| --- | --- | --- | --- | --- | --- |
| Age | — | –0.18 | –0.10 | 0.22 | 0.24 |
| OI | –0.18 | — | 0.68 | –0.45 | –0.62 |
| p(O₂) | –0.10 | 0.68 | — | –0.41 | –0.50 |
| p(CO₂) | 0.22 | –0.45 | –0.41 | — | 0.39 |
| p(A-a)O₂ | 0.24 | –0.62 | –0.50 | 0.39 | — |

All coefficients are Pearson’s r. No absolute correlation exceeded 0.70, and all variance inflation factor (VIF) values were < 5, indicating no severe multicollinearity.
